# More than a feeling?—Overruling the preoperatively templated offset option leads to a minor offset increase in short stem total hip arthroplasty

**DOI:** 10.1007/s00402-021-04331-y

**Published:** 2022-01-07

**Authors:** Matthias Luger, Christian Stadler, Rainer Hochgatterer, Jakob Allerstorfer, Tobias Gotterbarm, Antonio Klasan

**Affiliations:** 1 Department for Orthopedics and Traumatology, Kepler University Hospital GmbH, Krankenhausstrasse 9, 4020 Linz, Austria; 2 Johannes Kepler University Linz, Altenberger Strasse 69, 4040 Linz, Austria

**Keywords:** Short stem, Total hip arthroplasty, Digital templating, Femoral offset, Hip offset, Leg length

## Abstract

**Purpose:**

Short stems are increasingly used in total hip arthroplasty (THA) because of advantages in bone and soft tissue preservation and reconstruction of hip geometry. Digital templating is essential in determining the correct offset option and stem size in THA. However, the preoperative template sizes might be intraoperatively overruled.

**Patients and methods:**

We evaluated the effect of intraoperative overruling of the preoperatively templated offset option of a short curved stem on hip offset, leg length, implant positioning, and femoral canal fill index. The overruling was performed in case of intraoperative instability, telescoping, or both. A series of 1052 consecutive THAs with a cementless short curved stem and press-fit cup was retrospectively screened. One hundred patients with unilateral THA and a contralateral native and morphologically healthy hip as a reference met the inclusion criteria. Measurements were carried out on preoperative and 3 months anterior–posterior postoperative radiographs. Patients were divided according to the overruling by offset option or stem size.

**Results:**

Hip offset was increased in all groups, but only with significant increase if an offset option + 1 was used intraoperatively (*p* = 0.025). LLD was restored without significance in all groups (*p* = 0.323; *p* = 0.157).

**Conclusion:**

Intraoperative overruling of the preoperative digital template in cementless short stem total hip arthroplasty results in an increase of hip offset compared to a contralateral healthy hip. However, the increase is marginal and clearly under 5 mm compared to the contralateral healthy hip.

## Introduction

Short stem total hip arthroplasty (THA) is increasingly performed in recent years [1, 2]. Short stems have the advantage of superior preservation of the proximal bone stock [3], facilitate a more soft tissue conserving implantation in minimally invasive approaches [2, 4], and restore the hip geometry superiorly [5]. Conventional straight stems show excellent long-term outcomes [6], but have a limited ability to restore the femoral offset (FO) due to their straight geometry [5]. Besides leg length (LL), FO is an essential parameter for postoperative clinical functional outcome, dislocation rate, wear, and revision rate [5]. Restoration of the native FO increases postoperative range of motion, abductor muscle function, and decreases polyethylene wear [5, 7]. Several studies even suggest a beneficial effect of an increased FO on abductor muscle force and joint reaction [8, 9].

A successful postoperative outcome after total hip arthroplasty relies on restoring the biomechanics of the hip as well as selecting the appropriate implant size [10, 11]. The position of short stem implants is more variable and also depends on the femoral resection level and the anatomy of the femoral neck [12]. Therefore, preoperative templating is found as an integral part of selecting the correct implant size intraoperatively in order to avoid complications [13]. Undersizing can lead to component loosening, while oversizing can lead to intraoperative fractures [14, 15].

Preoperative templating in short stem THA is essential for determining the correct implant sizes in order to reconstruct the hip offset and restore the leg length adequately. While accuracy in digital templating for short stem THA is described as reliable compared to conventional straight stems [16], other studies suggest a lower rate of accuracy for short stems [17]. However, accurate digital templating is heavily dependent on a standardized and correctly applied imaging technique as well as the correct calibration of the digitalized radiography [18, 19]. In certain cases, surgeons may have to overrule the preoperative digital template in order to achieve sufficient joint stability. The effects on hip offset, leg length difference, and implant positioning in these cases of overruling preoperative templates in short stem THA are unknown. We therefore conducted this study to evaluate the effect on hip geometry, leg length, and implant positioning in THA with a cementless curved short stem with four different offset options.

## Methods

### Patients

This retrospective radiological comparative study includes patients of a consecutive series of THAs with the same cementless curved short stem (Fitmore^®^ stem, ZimmerBiomet, Warsaw, IN, USA) and bi-hemispherical press-fit acetabular cup (Allofit^®^/-S, ZimmerBiomet, Warsaw, IN, USA) performed via a minimally invasive supine anterolateral approach. Fitmore^®^ hip stem is a titanium alloy stem (Ti Al6V4) that has a porolock Ti-VPS coating in the proximal part to enhance bone ingrowth and is available in four different neck angle options (127°, 129°, 137°, 140°) and 14 different sizes (size 1–14) for each offset option [2]. A cementless titanium press-fit cup with or without screws (Allofit^®^/-S, ZimmerBiomet, Warsaw, IN, USA) was used in all patients.

A consecutive series of 1052 hips in 982 patients with index surgery between 2014 and 2019 were screened for inclusion and the medical records until 90 days postoperative were evaluated. Firstly, all patients were screened for existence of a preoperative digital templating. In 63 cases there was no preoperative template available. In a second step preoperative X-rays (both hips in comparison, anterior–posterior view, standing upright) of these 989 cases were screened for unilateral THA. Diagnoses for inclusion were primary osteoarthritis, avascular necrosis of the femoral head, or mild dysplasia of the hip (Crowe I) [20]. Exclusion criteria were defined as bilateral hip disease (Kellgren Lawrence > grade 1) [21], a history of prior hip surgery, previous trauma, postoperative complication, reoperation or revision for any reason, as well as missing pre- or postoperative radiographs. In total 100 patients met the inclusion criteria. Overruling the preoperative template was defined in different groups, Table 1. The offset of the Fitmore^®^ hip stem is primarily affected by the offset option itself and secondly by the stem, as an increasing stem size results also in an increased horizontal offset and vice versa.

**Table 1:** Description of the different overruled offset options and stem sizes

	Description	Number of cases (*n*)
Total		100
Offset 0	Offset option intraoperatively used as templated	67
Offset + 1	One offset option higher than templated used intraoperatively	21
Offset − 1	One offset option lower than templated used intraoperatively	11
Offset − 2	Two offset options lower than templated used intraoperatively	1
Total		100
Offset 0/Size 0	Offset option as templated used intraoperatively; Stem size used intraoperatively as templated	27
Offset 0/Size > + 1	Offset option as templated used intraoperatively; Stem size bigger 1 size or more used intraoperatively as templated	6
Offset 0/Size > − 1	Offset option as templated used intraoperatively; Stem size smaller 1 size or more used intraoperatively as templated	34
Offset ± 1 option	Other offset option as templated used intraoperatively	33

Radiographic measurements were performed on preoperative and 3-month postoperative low-centered anteroposterior (AP) radiographs of the pelvis in both groups. Preoperative age at operation, gender, body mass index (BMI), and laterality were recorded. The patient demographics are shown in Table 2 and were evaluated in two different groups: offset option intraoperatively used as templated (Group A) and any deviation in offset option implanted intraoperatively (Group B).

**Table 2:** Patient demographics, (Mean and SD), and Testing

Variable	Group A	Group B	*p* value
Number of Hips	67	33	–
Side (L:R)	31:36	15:18	1.000
Gender (F:M)	44:23	17:16	0.196
Age (years)	55.6 ± 10.5 (35–77.1)	56.8 ± 11.5 (27–75.2)	0.772
BMI at surgery	27.1 ± 4.1 (19.7–36.5)	29.3 ± 5.9 (20.5–47.3)	0.078

*SD* standard deviation, *F* female, *M* male, *L* left, *R* right, *BMI* body mass index (kg/m^2^)

This study was approved by the institutional review board (EK-No.: 1239/2019). Due to the retrospective study design with evaluation of pre-existing medical records, an informed consent was not required. All procedures performed in studies involving human participants were in accordance with the ethical standards of the institutional and/or national research committee and with the 1964 Helsinki declaration and its later amendments or comparable ethical standards.

### Digital templating

**Fig. 1:**
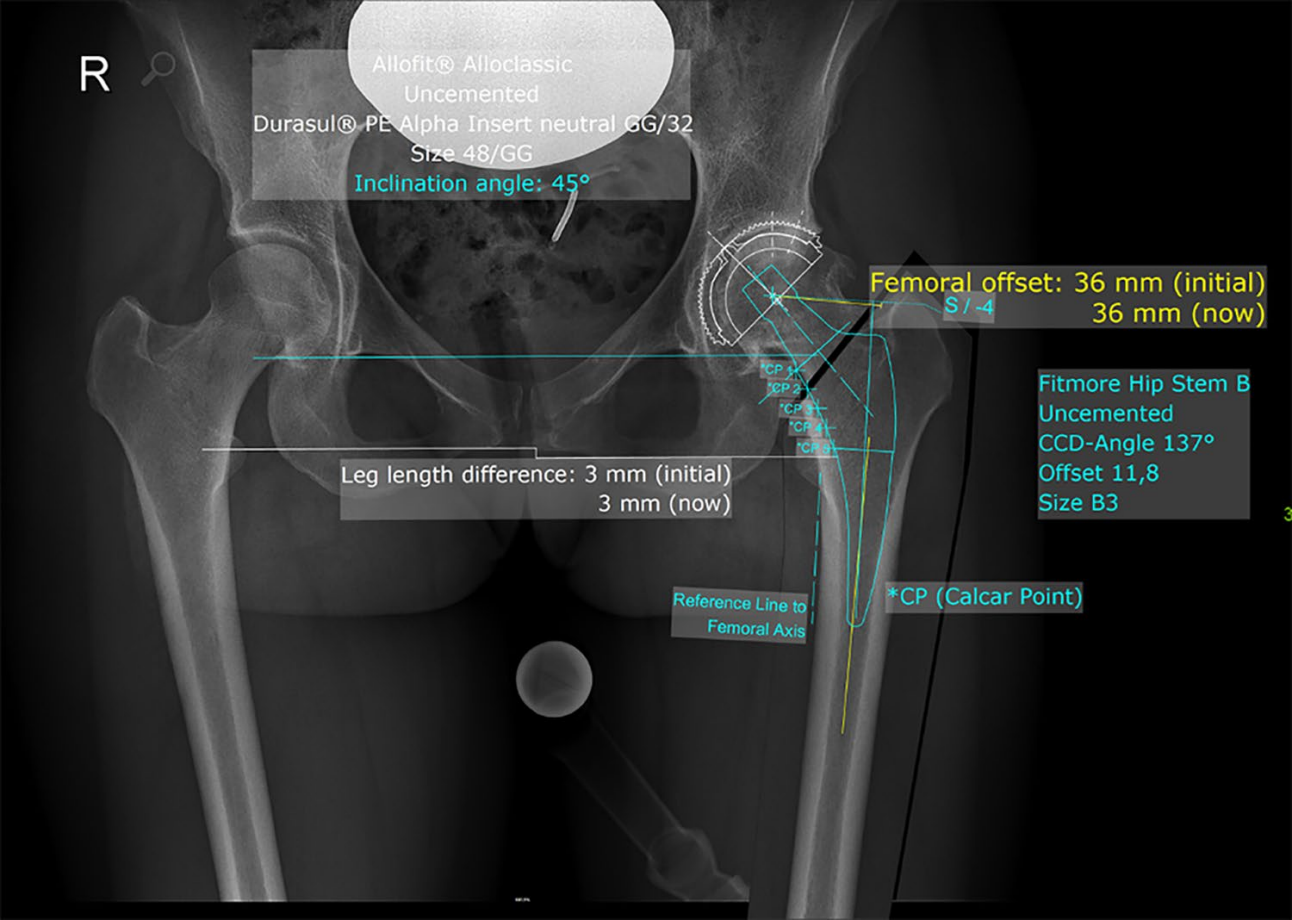
Preoperative digital template on an anterior–posterior radiograph of the pelvis

Digital templating was carried out with mediCAD^®^ version 5.1 (Hectec GmbH, Altdorf, Germany) in a standardized manner. Scaling was performed using a 25-mm metallic radiopaque ball as the reference, placed between the legs of the patient, aiming for the level of the hip in the frontal plane. The scaling was performed automatically by the software. Then, the center of rotation, the proximal femoral shaft axis, and the leg length discrepancy were determined. After that the correct size and position of the acetabular component were determined. Next the size of the femoral component was templated beginning with the correct offset option. The aim in templating a Fitmore hip stem is to restore the anatomical offset by confirming that the medial curve of the stem follows closely to the inner line of the cortex in the calcar region when the stem is in axis with the femoral canal. After choosing the correct offset option, the appropriate stem size is selected. The appropriate stem size is selected by choosing the stem which fills the intramedullary canal entirely. Figure 1 shows an example for digital templating as carried out in this study. In the presented study, the operating surgeon performed the preoperative digital template by a standardized templating procedure as explained above. However, the neck length was not templated in every case and was therefore not included for statistical analysis. All preoperative templates included cup size, stem offset, and stem size.

Overruling of the digital template leading to an increased offset was done by the operating surgeon in case of intraoperative necessity due to instability, "telescoping," or both after trial reduction. A higher offset option was chosen if stability could not be achieved by a higher head size offset. In case of increased of leg length difference discrepancy and instability or in case of impending leg length difference by using a higher head size, a higher offset option was chosen. This decision was performed at the discretion of the surgeon, based on intraoperative judgment and experience.

### Surgical technique and treatment protocol

Surgical procedures were carried out at the author’s institution by surgeons with different levels of experience, including 11 consultants and 7 residents. All consultants perform more than 50, all senior consultants more than 100 arthroplasties per year. Resident surgeries were done under the guidance of a consultant. In all cases a minimally invasive anterolateral Watson-Jones approach in supine position on a standard operating table under laminar air flow was performed. Extremity preparation was performed with threefold antiseptic scrub with alcohol disinfectant. Draping with a sterile adhesive surgical iodine film was used. The skin incision was centered over the greater trochanter. An incision at the border between the Tensor fasciae latae and the Tractus iliotibialis was performed. Then the Watson-Jones interval between Tensor fasciae latae and Gluteus medius was bluntly dissected. A capsulectomy was performed in every case. Fluoroscopy was routinely used with the definitive cup and trial stem in situ. The standardized peri- and postoperative protocol was identical in all cases, including single-shot antibiotics (Cefuroxime 1.5 g i.v. directly preoperative), weight bearing as tolerated from the first postoperative day on, Indomethacin 75 mg daily for the prevention of heterotopic ossification on days 1 to 4 postoperatively and 40 mg low-molecular weight heparin, or Rivaroxaban 10 mg for 28 days postoperatively as venous thromboembolic event prophylaxis.

### Radiographic evaluation

**Fig. 2:**
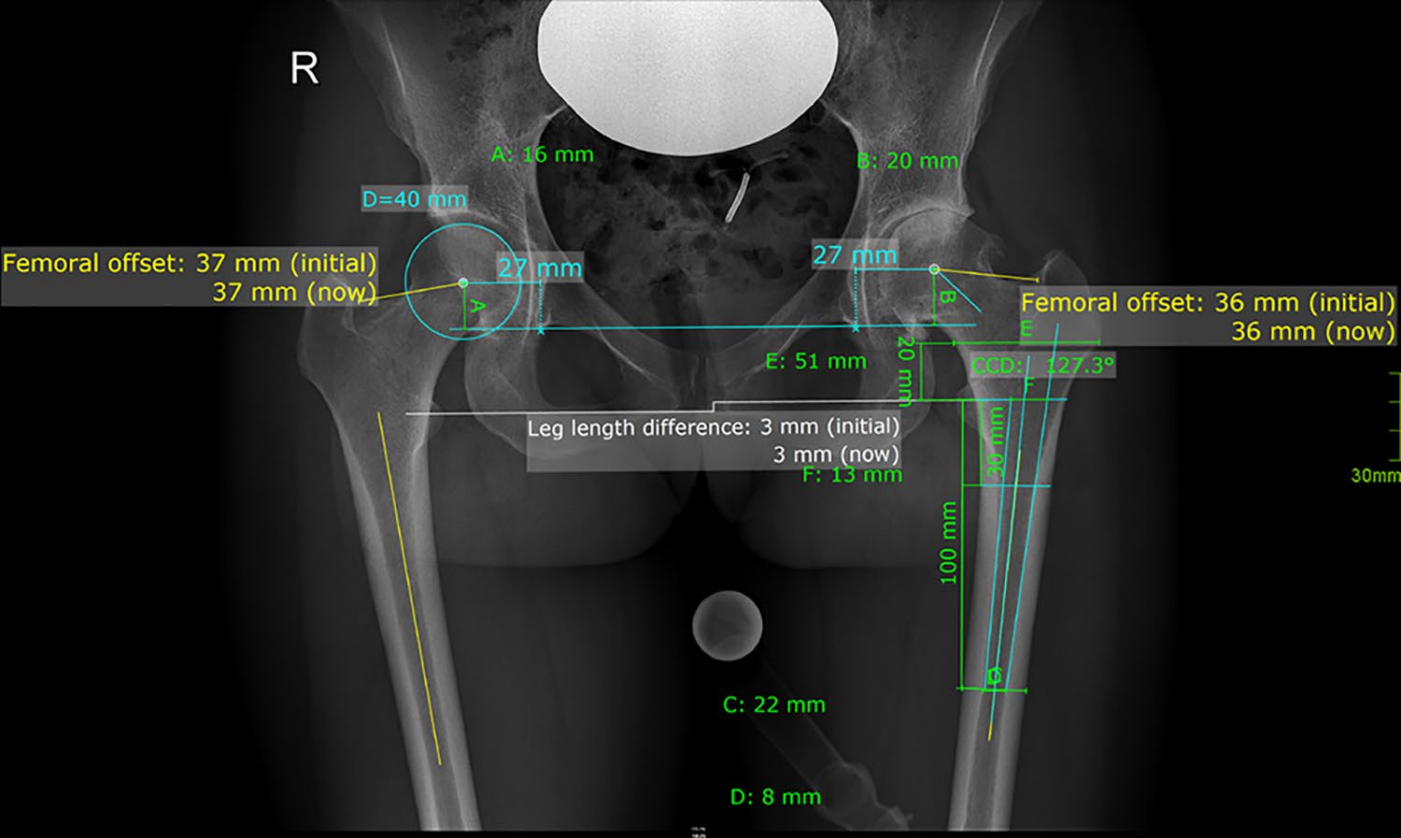
Preoperative measurements: Both sides: *FO* femoral offset, *AO* acetabular offset, *CCD angle* vertical position of the center of rotation (COR), Leg length difference (LLD); Affected hip: Centrum-Collum-Diaphyseal Angle, *CI* cortical index, *Canal Flare Index* Canal to Calcar ratio

Radiographic measurement was performed on preoperative and 3-month postoperative digital low-centered AP radiographs of the pelvis [22]. Measurement was conducted independently by two reviewers (M.L., J.A.), who were not involved in the index surgery. Radiographs were taken with the patient in standing position and with both legs in 15° internal rotation and the central beam was directed on the symphysis pubis [23]. In order to achieve an accurate measurement of the hip anatomy a double coordinate system was applied on both the preoperative and the postoperative images [1, 24]. Radiographic analysis was also performed using MediCAD^®^ Software V5.1. The hip center of rotation (COR) was defined using a circle tool determining the diameter of the femoral head and its center [25]. The femoral offset (FO) was determined as the perpendicular distance between the COR and the proximal femoral shaft axis (FSA) [22, 25]. Acetabular offset (AO) was measured as the perpendicular distance between the COR and line T, with T being the perpendicular line on the trans-teardrop line (TT) through the ipsilateral teardrop figure [22]. Hip offset (HO) was calculated as the sum of FO and AO [22]. The vertical position of the COR was measured as the perpendicular distance to line TT [26]. Radiographic leg length discrepancy (LLD) was measured as the perpendicular distance between line TT and the middle of the lesser trochanter (LT) [23]. Centrum-Collum-Diaphyseal (CCD) angle was determined according to M. E. Müller on the affected hip [27]. To characterize the anatomical shape of the proximal femur and the thickness of cortical bone, the canal to calcar isthmus ratio and the cortical index (CI) according to Dorr et al. [28] were determined. A high CI indicates a thick cortical bone [28]. Additionally the canal flare according to Noble et al. [29] was determined. On preoperative X-rays FO, AO, HO, LLD, and vertical position of the COR were measured bilaterally, while CCD angle, CI, Canal Flare Index, and Canal to Calcar Ratio were measured unilaterally on the affected hip. Complete preoperative measurements are also shown in Fig. 2.

**Fig. 3:**
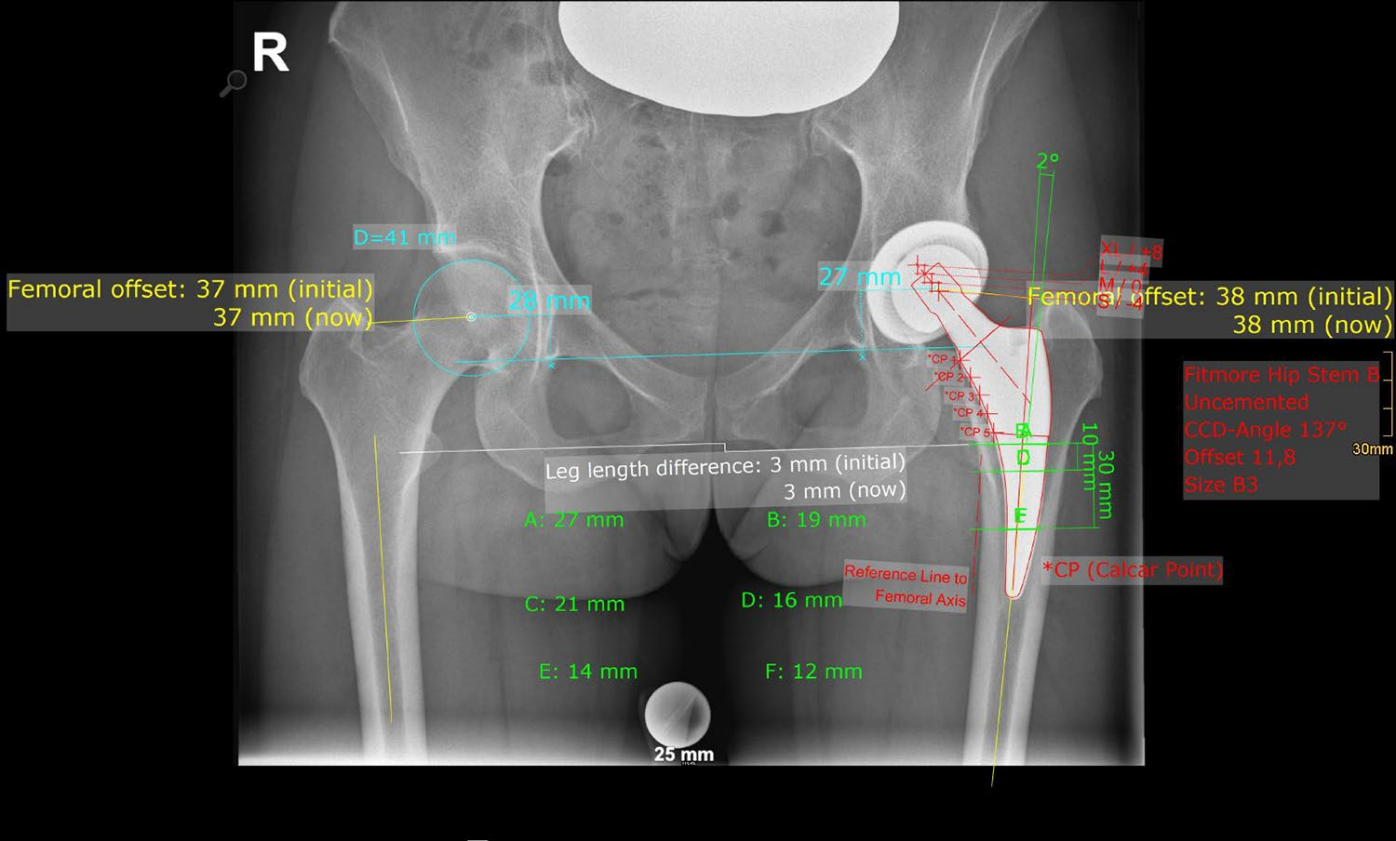
Both sides: *FO* femoral offset, *AO* acetabular offset, *COR* vertical position of the center of rotation, *LLD* Leg length difference

On postoperative X-rays FO, AO, HO, LLD, and vertical position of the COR were measured bilaterally. Complete postoperative measurements are also shown in Fig. 3.

Intra- and interobserver reliabilities were calculated for 15 randomly selected cases for each group. Intraclass correlation coefficients (ICC) were used with a two-way random effects model for absolute agreement. Repeated measurements for intraobserver reliability were performed at day 1 and day 14 in a blinded fashion.

### Statistics

Descriptive statistical analysis was conducted for age, gender, body mass index (BMI), and laterality. A Shapiro–Wilk test was performed for testing normal distribution. As not all variables were normally distributed non-parametric testing was performed. For patient demographics a Fisher’s exact test was performed on categorical variables (gender and laterality). A Wilcoxon–Mann–Whitney *U* test was performed on continuous variables (age and BMI). For statistical analysis of pre- and postoperative radiographic measurements, a non-parametric Kruskal–Wallis test was performed. Power analysis was not performed due to the observed statistical significance [30]. Therefore, the null hypothesis was to find out that overruling the preoperative digital template in total hip arthroplasty does not affect hip offset compared to the contralateral healthy hip. Statistical analysis was calculated with SPSS version 26 (IBM SPSS statistics, Chicago, IL, USA). A *p* value < 0.05 was considered as statistically significant.

## Results

The interobserver and intraobserver correlation coefficient for radiographic measurements showed satisfying results (range, 0.961 [95% CI, 0.853–0.989] to 0.998 [95% CI, 0.986–0.999]). Average age at operation, gender, BMI, and laterality were evenly distributed and did not show any statistical significance as shown in Table 3. Difference in HO, AO, FO, LLD, and vertical position of the COR did not show any statistical difference in both groups as shown in Table 3. Only the CCD angle showed statistically significant differences, Table 3. Anatomical shape of the affected side did not show any significance in testing of the Cortical Index, Canal Flare Index, and Canal to Calcar ratio.

**Table 3:** Preoperative radiographic measurements

Preoperative discrepancy between arthritic and healthy hip	Offset 0	Offset -1	Offset + 1	Offset − 2	*p* value
Hip offset (mm)	1.5 ± 3.8	0 ± 4.7	0.9 ± 4.7	− 3	0.286
Acetabular offset (mm)	0.5 ± 2.5	2.5 ± 3.2	0.7 ± 1.7	1.00	0.298
Femoral offset (mm)	2 ± 3.9	2.5 ± 5	1.6 ± 4.5	− 2	0.622
Leg length difference (mm)	− 4.1 ± 4.4	− 3.4 ± 6.7	− 2.7 ± 5	4.00	0.323
Vertical position of the COR (mm)	2.9 ± 3.9	0.6 ± 4.2	2.5 ± 2.8	− 2.00	0.119
CCD angle (°)	131.9 ± 6.7	126.9 ± 5.5	131.6 ± 5.8	119.8	**0.049**
Cortical Index	0.6 ± 0.04	0.62 ± 0.04	0.69 ± 0.06	0.66	0.302
Canal Flare Index	4.66 ± 0.7	4.84 ± 0.64	4.76 ± 1.01	5.4	0.426
Canal to Calcar ratio	0.62 ± 0.1	0.6 ± 0.09	0.62 ± 0.09	0.63	0.965

All values are expressed as mean ± standard deviation

*COR* center of rotation, *CCD angle* centrum-collum-diaphyseal angle

Significant values in bold letters

Table 4 shows the postoperative measurements in detail. Postoperative HO only increased if an offset option + 1 option was used (*p* = 0.025). Apart from group “Offset -2” AO was reduced in all groups, Table 4. FO was increased in all groups, Table 4. The vertical center of rotation was cranialized significantly in all groups, Table 4. The LLD did not show significant differences between the groups, Table 4.

**Table 4:** Postoperative radiographic measurements and testing for statistically significant differences between the operated side and the contralateral healthy hip

	THA	Healthy hip	*p* value
Hip offset (HO) (mm)			
Offset 0	74 ± 9	71.9 ± 7.8	0.405
Offset + 1	77.6 ± 7	73.4 ± 5.9	0.025
Offset − 1	77.55 ± 11.3	74.7 ± 10.5	0.276
Offset − 2	84	78	−
Offset 0/Size 0	75.8 ± 10.6	72.9 ± 8.7	0.562
Offset 0/Size ≥ + 1	75.3 ± 13.2	72.7 ± 12.6	0.575
Offset 0/Size ≥ − 1	72.3 ± 6.5	70.9 ± 6	0.468
Offset ± 1 option	77.8 ± 8.5	74 ± 7.5	0.035
Acetabular Offset (AO) (mm)			
Offset 0	29.3 ± 3.3	33.1 ± 4	< 0.001
Offset + 1	28 ± 4.1	33.2 ± 3.9	< 0.001
Offset − 1	30.6 ± 3.5	31.6 ± 5.7	0.408
Offset − 2	31	31	−
Offset 0/Size 0	29.1 ± 2.8	33.1 ± 4.2	< 0.001
Offset 0/Size ≥ + 1	31 ± 4.7	35.3 ± 6.3	0.227
Offset 0/Size ≥ − 1	29.2 ± 3.3	32.7 ± 3.3	< 0.001
Offset ± 1 option	29 ± 4	32.6 ± 4.5	< 0.001
Femoral Offset (FO) (mm)			
Offset 0	44.7 ± 8.5	38.8 ± 6	< 0.001
Offset + 1	49.6 ± 6.3	40.1 ± 5.1	< 0.001
Offset − 1	46.9 ± 9.5	43.1 ± 5.4	0.167
Offset − 2	53	47	–
Offset 0/Size 0	46.7 ± 10.2	39.8 ± 6.3	0.014
Offset 0/Size ≥ + 1	44.3 ± 10.6	37.3 ± 6.5	0.334
Offset 0/Size ≥ − 1	43.2 ± 6.4	38.2 ± 5.6	0.003
Offset ± 1 option	48.8 ± 7.4	41.3 ± 5.3	< 0.001
Vertical position of the COR (mm)			
Offset 0	19.1 ± 3.4	14 ± 4	< 0.001
Offset + 1	20.9 ± 5	15.6 ± 3.5	< 0.001
Offset − 1	16.8 ± 3	14.1 ± 1.8	0.021
Offset − 2	15	12	–
Offset 0/Size 0	19.2 ± 2.6	13.7 ± 3.5	< 0.001
Offset 0/Size ≥ + 1	19.8 ± 3.5	15.2 ± 3.8	0.045
Offset 0/Size ≥ − 1	18.9 ± 4	13.9 ± 4.4	< 0.001
Offset ± 1 option	19.3 ± 4.7	15 ± 3.1	< 0.001
LLD (mm)			
Offset 0	− 0.3		0.323
Offset + 1	− 0.3		
Offset − 1	− 0.1		
Offset − 2	6		
Offset 0/Size 0	0.3 ± 5.2		0.157
Offset 0/Size ≥ + 1	0.7 ± 3.6		
Offset 0/Size ≥ − 1	− 0.9 ± 5.6		
Offset ± 1 option	0.0 ± 5		

## Discussion

The results of this present study show minor increase in HO regardless of following or overruling the preoperative digital templating. Additionally, leg length difference was also without any statistically significant difference in both groups. Accurate reconstruction of hip geometry in THA is essential and has influence on clinical outcome, dislocation risk, range of motion, impingement, abductor muscle strength, and polyethylene wear [31–33]. The impact of offset reconstruction on the clinical outcome has been extensively examined. Innmann et al. [34] reported the best improvement in clinical outcome with a combination of complete to slightly increased HO (± 5 mm) reconstruction and a marginal LLD in short stem THA with Fitmore^®^ hip stem. Mahmood et al. [31] reported weaker hip abductor muscle strength in patients with a decrease in HO by more than 5 mm. Sariali et al. [33] reported comparable findings with altered gait with asymmetry between both hips, reduced range of motion, and a lower maximal swing speed on the operated side for patients with a minimum decrease in FO of 15%. Cassidy et al. [9] reported that a decrease in FO of more than 5 mm resulted in worse Western Ontario and McMaster Universities Arthritis Index (WOMAC) scores compared to patients with reconstructed or increased FO. Our results show an increase of HO in several groups compared to a healthy contralateral hip. However, a significant increase of HO was observed in cases of an overruling by one offset option higher than templated (*p* = 0.025). In all other groups HO was increased without significant difference to the contralateral healthy hip. An increase in HO of ≥ 5 mm compared to the contralateral normal hip negatively affects polyethylene wear [32]. We report values in a range under an increase of 5 mm for all groups, which is suggested to be superior for clinical functional outcome [34] and for polyethylene wear [32].

Overruling the preoperative digital templating did not pose a risk for leg length difference. Adequate reconstruction of HO and LL is considered as clinically important in THA [22, 34]. However, the literature on leg length difference after THA and its clinical influence are inconsistent [34]. The consensus agreement recommends a LLD, that is kept to a minimum [31, 35]. In all groups leg length was restored without significant differences independently of following or overruling preoperative templating.

Successful preoperative digital templating also relies on the correct X-ray and its quality. Merle et al.[25] described a risk of underestimation of FO in AP pelvis X-rays with an average of 13% compared to CT scans. In case of male patients, the effect could be additive because of averagely higher FO. Therefore, the preoperatively templated offset option and size might be too small because of the used X-ray. In this study, AP X-rays of the pelvis were used. Therefore, the use of a different offset option intraoperatively was necessary, because of an underestimation on the preoperative template.

Several limitations of the study have to be addressed. Firstly, we tried to minimize a potential selection bias with very strict inclusion criteria. We present a consecutive cohort with over 1000 THAs, which was reviewed for inclusion. Only patients with a single implant design and approach were included. A homogeneous study cohort was created by excluding patients with a bilateral hip disease (Kellgren Lawrence > grade 1). Both study groups were tested for differences in age at surgery, BMI, laterality, and gender without any significance. Also, preoperative measurements were tested without any statistically significant differences. Furthermore, we aimed to increase reliability of the measurements and results by restricting inclusion based on preoperative diagnosis. We excluded all forms of secondary osteoarthritis of the hip and development dysplasia of the hip Crowe grade II to IV. Prior surgery before THA was also excluded. However, mild hip dysplasia (lateral center–edge angle 20°–25°), coxa profunda, and morphologic alterations related to cam- or pincer-type impingement were included, because these changes might be subtle and cannot be reliably identified in the present study cohort with end-stage disease. Therefore, we conclude that the findings in the present study are applicable for primary osteoarthritis and care must be taken when applying our findings on secondary osteoarthritis or high grades of development dysplasia of the hip. Secondly, we address the fact of taking measurements on plain radiographs. FO is underestimated by approximately 13% on plain radiographs [25]. Additionally, radiographic measurement of leg length difference does not necessarily reflect clinical leg length difference [36]. However, our measurements are easily reproducible, applicable in daily routine, and less invasive regarding radiation exposure. Furthermore, we postulate variances in inter- and intraobserver reliability in measuring clinical leg length difference. We acknowledge the restrictions of measurements on plain radiographs. But with implementing strict inclusion criteria and by using reproducible and well-described landmarks for measuring, we postulate a sufficient reduction of these limitations.

## Conclusion

Intraoperative overruling of the preoperative digital template in cementless short stem total hip arthroplasty results in an increase of hip offset compared to a contralateral healthy hip. However, the increase is marginal and clearly under 5 mm compared to the contralateral healthy hip.

## Data Availability

Data and materials are available on request.
